# The Use of Online CB-ART Interventions in the Context of COVID-19: Enhancing Salutogenic Coping

**DOI:** 10.3390/ijerph18042057

**Published:** 2021-02-20

**Authors:** Dorit Segal-Engelchin, Ephrat Huss, Orly Sarid

**Affiliations:** Spitzer Department of Social Work, Ben- Gurion University of the Negev, P.O. Box 653, Beer-Sheva 84105, Israel; ehuss@bgu.ac.il (E.H.); orlysa@bgu.ac.il (O.S.)

**Keywords:** online CB-ART intervention, COVID-19-related stress, coping resources, salutogenic continuum, mental health professionals

## Abstract

Community crises require the provision of short-term reflective intervention methods to help service users identify stressors, and access and intensify their adaptive coping. Here, we demonstrate the use of a single-session online cognitive behavioral- and art-based (CB-ART) intervention within the context of the COVID-19 pandemic. In this method, the individual draws three images: his/her COVID-19-related stress, his/her perceived resources, and an integration of stress and resources. This method provided a reflective space in which individuals could identify their experienced stressors, acknowledge their coping resources, and integrate these two elements within the context of the current pandemic. In this article, we use illustrative examples from a study implemented during the first national lockdown in Israel and present a tool that can be easily implemented by mental-health professionals in ongoing community crises. The aims of this intervention were to co-create knowledge with service users, access their self-defined needs and strengths, and enhance their coping by enabling them to view stress and coping as part of the salutogenic continuum.

## 1. Introduction

Throughout the coronavirus disease 2019 (COVID-19) pandemic, individuals and communities have had to cope with ongoing distress caused by lockdown, social distancing, social isolation [1], and other personal stressors [2]. It is important to understand people’s phenomenological experience of this crisis, including the emerging and shifting stressors and evolving coping strategies. For example, one may initially suffer loneliness in lockdown, which may be followed by financial problems due to unemployment. These shifting and unforeseen stressors demand constant re-evaluation of both the phenomenological experience of stress and the coping strategies [3,4,5,6,7].

The predominant medical model of stress and trauma focuses on measuring distress and symptoms. However, in long-term community crises, ongoing strategies are needed to define changing stressor levels and types, as well as enhance self-coping [8,9]. Crises require a rapid understanding of how people experience stressors, then identifying and encouraging coping resources. Enhancing one’s sense of mastery and skills to battle distress and increase one’s resilience are major and ongoing tasks in long-term crises [10,11,12,13]. Stress and coping are often treated as fully separate areas. However, it is important to contextualize both stress and coping as interactive and as playing out within a specific phenomenological and contextual reality [11,14,15]. In Antonovsky’s [16] concept of salutogenesis, stress and coping are a continuum that constantly interact [17,18], thus breaking the dichotomy between health and disease. According to Antonovsky [17], individuals have overall resources and resilience strategies that help them conceptualize the world as comprehensive, understandable, and manageable. Compared to a person lacking this belief, an individual who believes that she/he has the resources needed to deal with a stress-related situation can find meaning and motivation to cope with it in a more resilient way.

Existing literature has discussed the potential of arts to depict and excavate stress, enhance resilience [12,19,20,21,22], and situate phenomenological experience within specific contexts [14,23]. Art enables depiction of how stress emerges from society into the individual, and this understanding constitutes the first step in addressing it [10,11,14]. In terms of the salutogenic theory, the arts help excavate comprehensibility and meanings, while also embodying manageability. Indeed, the arts can enable symbolic “management” of a crisis on the page through actively expressing and defining it. Further verbal elaboration within a group enhances comprehension and meaning levels. Integrating a stress image with a coping image can be considered an embodiment of the central salutogenic concept of creating a continuum between stress and coping [12,16,24].

Community crises require short-term, focused, and reflective intervention methods to help service users identify stressors, which access and intensify their adaptive coping [3]. Arts-based methods enable access and organization of embodied images of stress, and the ignition of new perceptions and creativity towards managing that stress [13]. The arts can re-ignite creative ways of addressing old problems with new perceptions [19,22,25,26]. Images contain content that is intensely relevant to the individual, and the details enable further elaboration of meanings and access to how concrete subjective experiences can be transformed [21,23,27,28,29]. A person can excavate their internal narrative through decisions regarding the shapes, colors, and placements of the elements of his/her narrative, intensifying the reflective and expressive elements of the expressions of stress and coping [11,23,25].

Previous studies demonstrated that cognitive behavioral- and art-based (CB-ART) interventions effectively reduced stress related to community crises (e.g., wars [30] and earthquakes [31]). Cognitive behavioral interventions and art therapy stem from different theoretical orientations, yet both entail a multi-faceted intervention that creates flexible pathways between the physical, emotional, and cognitive aspects of a stressful event. Moreover, both intervention types combine physical and emotional stress reduction techniques through the use of images [13]. Together, they offer a unique tool for image transformation as a strategy for managing distress in extremely stressful situations. While prior CB-ART interventions were performed face-to-face in group settings, here we demonstrate their application online via Zoom in a group setting. Information and communication technology can provide a venue for communicating about stress and coping mechanisms [32,33], and serve as a platform for digital psychological interventions [34,35].

In this article, we demonstrate a CB-ART intervention for stress reduction during the COVID-19 pandemic, using illustrative examples from a study conducted during the first national lockdown in Israel [36]. Within the intervention, participants drew three images: their experienced stress, their perceived resources, and an integration of the stress and resources. Our aim is to provide mental health professionals with this simple CB-ART intervention for application during community crises. This tool can be used online as an ongoing evaluative tool, short-term intervention, and group work method for accessing and integrating stress and coping, using both micro and macro perspectives to deal with the challenges of a long-term community crisis situation in a shifting socio-cultural context.

## 2. Materials and Methods

We examined a CB-ART intervention that was implemented during the first national lockdown [36]. In accordance with the social distancing restrictions in Israel during the COVID-19 pandemic, two CB-ART workshops were conducted via Zoom. The workshops were conducted by the authors, whose areas of expertise include cognitive behavioral- and arts-based interventions. Participants were recruited using a snowball sampling technique based on a post published on Facebook. They were contacted by phone, provided information regarding the study objective and procedure, and told that they could participate in the workshop regardless of whether they volunteered for the study. All workshop participants (N = 15) volunteered for the study. They were asked to sign and return by e-mail a form consenting to the use of their drawings and questionnaire in the study. The participants were all females. They had an average age of 39 years (SD, 6 years). The majority were married (73.3%) and had an academic degree (80%).

Before the workshop, participants were instructed to complete and return by e-mail the first part of the study questionnaire, which included demographic information and a question regarding their distress level. Participants were asked to assess their level of distress using the 11-point Subjective Units of Distress Scale (SUDS) [37], ranging from 0 (absence of distress) to 10 (extreme level of distress). The three-hour workshop opened with a brief introductory lecture about stressful situations; stress-related reactions; and how negative distressing images, symptoms, or memories can have debilitating effects on mood. We explained that drawings can be analyzed in terms of both the narrative ascribed to them and their compositional elements, such as the shape, size, colors, and placement of the images on the paper.

In terms of art instructions, we followed a protocol previously used for CB-ART interventions [8,13,30,31], which included three drawings. The first was the stress drawing, for which the participants were guided to draw their current stress (image, symptom, or memory) and experiences linked to the COVID-19 pandemic. The second drawing was the resources drawing, for which the participants were instructed to draw a picture presenting their personal and social resources that could assist them in managing stressful situations during the COVID-19 pandemic. Finally, the third was the integrated drawing, for which participants were instructed to draw a picture that integrated their previously illustrated stressors and coping resources into a single image. Participants were given the option to draw a new picture or add elements to either their stress drawing or resources drawing. On the back of each drawing, the participants were asked to write a brief description of their artwork.

After completing each drawing, the participants presented their artwork within the group setting, they described what they had drawn, and shared their narrative. Then the group discussed the compositional characteristics of each drawing. The stress drawing enabled participants to identify the sources of their COVID-19-related stress, while the resources drawing helped participants identify their available coping resources. In the discussion of the integrated drawing, the group further compared the compositional elements to those in the stress and resources drawings. The integrated drawing was designed to “build bridges” between the first two drawings by creating an integrated image that combined both stressors and resources to help participants gain a sense that they can better cope with the stressors.

At the end of the workshop, participants were instructed to complete and return by e-mail the second part of the study questionnaire, which included questions addressing the compositional elements that they had used in the stress drawing and the transformations of those compositional elements within their integrated drawing. Participants also were asked to note their post-intervention distress level using the SUDS [37]. Finally, they were instructed to scan or photograph their drawings and the description of their artwork, and to send these images by e-mail to the research team.

To enable comparisons of the three drawings on an individual level, participants were asked to provide the last four digits of their national ID number on the questionnaire and the three drawings. They were told that this information was required for research purposes and would not be used to identify them. Participants were ensured that upon completion of data collection, their e-mails and e-mail addresses would be deleted from the researchers’ e-mail system. This study was approved by the social work departmental ethics committee at Ben-Gurion University of the Negev.

## 3. Illustrative Examples of the Drawings

Here, we present three illustrative examples of drawings and explanatory narratives from the above-described study [36].

**Figure 1:**
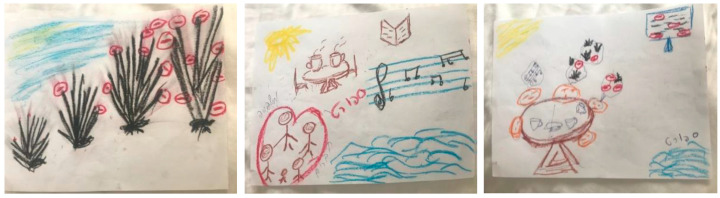
Decreasing the size of the stress image and giving more place to the resources within the integrated drawing.

The drawings in the first example were created by a 61-year-old married woman with an academic degree. In this example, the stress image (Figure 1, left) was explained by the participant as showing a scared hedgehog that puffs out its spines. The spines are blocked by all of the social distancing restrictions due to COVID-19 (symbolized by red stop signs). The hedgehog grows bigger and bigger in parallel with the spread of the pandemic and the increased restrictions. The resource image (Figure 1, center) includes the participant’s family and elements of leisure activities, such as sports (manifested by the word), listening to music, reading, visiting the beach, and having coffee with a loved one. This drawing displays both internal and external resources and meaning is provided by both family and cultural elements. In the integrated drawing (Figure 1, right), the most prominent image is of the family members conversing over a meal while hearing COVID-19 updates from a television in the background. Each family member is occupied with his/her own thoughts, as symbolized by smaller hedgehogs and music notes.

In explaining the compositional changes within the integrative drawing, this participant noted that in the integrated drawing she had added objects and additional colors (black was a dominant color in the stress drawing), decreased the hedgehogs’ sizes, and gave more space to the resources. The narrative ascribed to the integrated drawing indicates that this participant considers engagement in family activities, such as having a conversation over a meal, as a major stress-reducing strategy. It appears that the thought of being in a supportive family environment enabled her to perceive the pandemic-related stressors as less threatening, as reflected by the decreased hedgehog size and the greater space occupied by resources in the integrated drawing. This participant reported pre- and post-intervention distress scores of 7 and 4, respectively.

**Figure 2:**
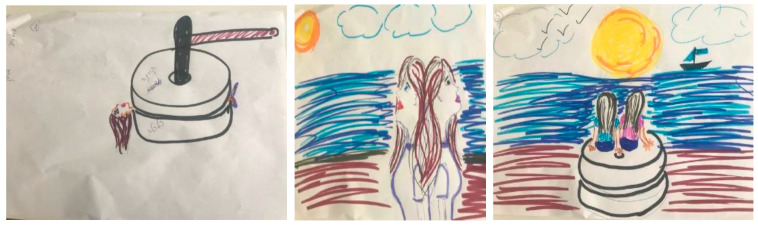
Placing the stress image in a secure context within the integrated drawing.

The drawings in example 2 were created by a 44-year-old single woman with a high school diploma. In this example, the stress drawing (Figure 2, left) includes an image of a millstone. This participant wrote that she drew herself trapped between the financial problems that she faced due to the shutdown of her business during the lockdown (symbolized by the lower stone), and her health concerns due to the spread of the virus (symbolized by the upper stone). When presenting her drawing, she emphasized her sense of helplessness while stuck between these two images. The drawing reflects her view that the crisis is eroding both financial stability and health, and that the intensity of these stressors erodes her body. In her resources drawing (Figure 2, center), this participant explained that she drew the resources that enhance her strengths in stressful situations, including the beach, the sun, and particularly her sister, who she calls her “almost twin sister” and to whom she feels very close.

The integrated drawing (Figure 2, right) presents the millstone with the beach and her major social resource—her sister. The context of the millstone has shifted compared to in the stress drawing—as it is now placed at the beach which she perceives as a calm and secure place. In the integrated drawing, she and her sister are sitting together on the millstone, indicating that together they can manage the stressors. They are both watching flying birds, symbolizing freedom of movement and the ability to fly. With regards to the compositional elements, the stress drawing had one dominant figure, while the integrative drawing includes additional elements—such as figures, colors, and lines—and a diminished size of the stress-related image. This participant reported pre- and post-intervention distress scores of 6 and 2, respectively.

**Figure 3:**
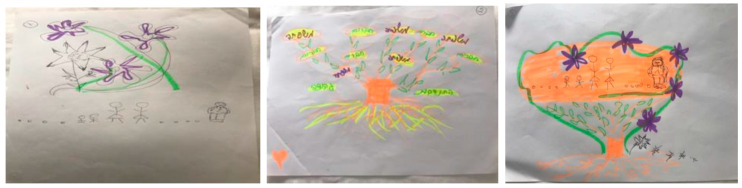
Altering the placement and size of the stress image within the integrated drawing.

The drawings in example 3 were created by a 60-year-old married woman with an academic degree. In this example, the stress drawing (Figure 3, left) shows flowers wrapping a thorn, representing pleasant things that occurred during the pandemic (e.g., increased family time due to the lockdown). The participant explained that “the flowers above the family members and friends, who are presented at the bottom of the drawing, provide them with a sense of calmness.” This stress drawing expresses only mild distress, possibly reflecting the participant’s attempt to distance herself from the stress associated with her pandemic-related experiences. In her resources drawing (Figure 3, center), the participant drew an image of a tree that displays the various resources that help her to manage stressful situations—her roots, family, friends, workplace, and engagement in volunteering activities—all manifested by words on the tree’s branches. The participant also noted that the feeling of being loved, represented by the heart in the lower left corner, is an important resource that helps her cope with stress. Her manageability and meaning are to help and support others, which enables her to deal with the stress.

This participant explained her integrated drawing (Figure 3, right) as follows: “the flowers surrounding the tree provide a sense of calmness that begins to wrap my loved ones, and the thorns are gradually shrinking in size till they completely disappear.” Within the integrated drawing, the stress image was altered in placement and size. In the stress drawing, the thorn representing the coronavirus appears above the participant’s loved ones. In contrast, in the integrated drawing, the participant’s loved ones are placed above the stressful image. This participant reported pre- and post-intervention distress scores of 5 and 2, respectively.

## 4. Discussion

The aim of this article was to provide a method for accessing service users’ self-defined experiences of stress and coping, as well as to help integrate these experiences into a continuum. A long-term community crisis creates chains of ongoing stressors that people cope with in different ways. Here, we demonstrated how a simple art activity, CB-ART, can be used to integrate stress and coping, and integrate phenomenological experience and social reality—elements that are often separated in different disciplines.

In our illustrative examples, we show different ways that people integrated their stressors and coping. In example 1, when the participant integrated the stress element into the resources image, the stressor did not disappear but was reduced in size and impact. It was placed alongside the resources that were the focus. This perspective enabled the participant to “live with” the stressor, rather than dissociate from it or be overwhelmed by it. It seems that manageability was attained by fully identifying the stressor, then fully identifying the most meaningful resources. For this participant, the resource image became the background to the stressor, rather than vice versa. In example 2, the participant displayed her extremely stressful situation of being squashed in a millstone between financial and health concerns. She managed this stress by utilizing her sister’s physical social support, which enabled her to shift her placement relative to the stressor. The stress remained at the center of the image but with she and her sister sitting together on top of it—illustrating that she cannot be crushed by it when they are together. In example 3, the participant chose to focus on coping already in the stress picture. It is possible that this participant found exposure to COVID-19-related stress to be so frightening that it could not be drawn alone. In this participant’s resources drawing, helping others emerged as a central coping resource.

The above examples illustrate how people may find different ways of integrating the spectrum of stress and coping—e.g., juxtaposing the stress into the coping resources, or the coping resources into the stress; shifting the size, placement, or intensity of the stress or the coping resources to create a better balance. Manipulating different compositional elements in the integrative drawing enables the integration of stress and coping in interactive and creative new ways [28]. These elements could continuously be re-shuffled in response to the shifting challenges of the ongoing crisis in terms of both reality and their phenomenological experience. The use of art to excavate coping and stressors, and to then integrate them in different ways, enables one to focus in on this process.

Art can also be used to enhance salutogenic coping. The dual levels of drawing and then explaining one’s drawing enable further solidification of a coherent narrative of the experience, enhancing meanings and comprehensions with which to cope with the stressful situation [12]. This can also be influenced by the observer(s) of the image. Stress often makes it difficult to think of inner or outer resources, and a person may become avoidant, flooded, or rigid [19]. Creativity can help dislodge this rigidity by enabling states of flow [38]. In a long-term crisis, stressors are likely shifting and also long-term; thus, they must be incorporated into daily life (e.g., continuing financial and health stress). Drawings can help a person plan for the future by determining how she/he personally experiences a social stressor and how she/he can apply specific inner and outer resources to this stressor, thus intensifying the element of manageability.

The presently described arts-based tool can be utilized in different ways by mental health professionals. It can be a means of co-creating knowledge with a service user [29], a short-term and motivational intervention focusing on strengths and coping, and a form of self-care to reduce the stress of mental health professionals in shared reality contexts [3,30]. The intervention can be conducted alone and quickly and can help contain contents within group work. Verbal sharing can help reduce isolation and group members can suggest additional strengths. However, it must be noted that mental health professionals implementing the CB-ART intervention must have basic knowledge of cognitive-, behavioral-, and arts-based interventions. This tool can also be applied for researching how different populations experience interactions between stress and coping [28]. Importantly, this intervention can easily be performed online, and creates a more embodied and interactional space to address the crises. This helps participants access senses and intensify online communication, which is often a relationally cold or disembodied verbal experience.

The major limitation of the above-described study [36] is the relatively homogeneous sample, which was mainly comprised of educated married women. Further research in more heterogeneous samples may advance our understanding of the different ways that people of diverse backgrounds integrate their stressors and coping, and enhance their salutogenic coping within the context of a community crisis.

## 5. Conclusions

The presently described CB-ART intervention provides mental health professionals a simple method for ongoing collaborative exploration of the self-defined stressors and strengths that people experience during an ongoing crisis. Rather than encapsulating stress and coping as separate phenomena, this method promotes our understanding of them as dynamic, synergetic, and interactive. The art-based tool can be easily implemented by mental-health professionals in on-going community crises, to help service users identify their experienced stressors and available coping resources, and to enable them to integrate them into a synergetic gestalt based within a specific socio-cultural context. This, in turn, may enhance the service users’ coping abilities by enabling them to view stress and coping as part of the salutogenic continuum.

## Data Availability

The data presented in this study are available in the article. The Use of Online CB-ART Interventions in the Context of COVID-19: Enhancing Salutogenic Coping.
